# The complete chloroplast genome of *Sauvagesia rhodoleuca,* an endangered species endemic to China

**DOI:** 10.1080/23802359.2021.1951141

**Published:** 2021-07-15

**Authors:** Xiao Xie, Jiashuang Huang, Yonghua Zhang, Shanshan Zhu

**Affiliations:** a School of Marine Sciences, Ningbo University, Ningbo, China; b College of Life and Environmental Sciences, Wenzhou University, Wenzhou, China; c Systematic & Evolutionary Botany and Biodiversity group, MOE Laboratory of Biosystem Homeostasis and Protection, College of Life Sciences, Zhejiang University, Hangzhou, China

**Keywords:** *Sauvagesia rhodoleuca*, chloroplast genome, phylogenomics

## Abstract

*Sauvagesia rhodoleuca* is an endangered and national key protected species of China, with limited natural distribution in Guangdong and Guangxi, Southern China. Here we reported the first complete chloroplast genome of *S. rhodoleuca* using genome skimming approach. The chloroplast genome is 157,300 bp in length, with a large single-copy region (LSC) of 86,021 bp and a small single-copy region (SSC) of 18,137 bp separated by a pair of inverted repeats (IRs) of 26,571 bp. It encodes 112 unique genes, including 80 protein-coding genes, 28 transfer RNA genes, and four ribosomal RNA genes. Phylogenetic analysis results strongly supported that *S. rhodoleuca* was closely related to *Medusagyne oppositifolia.*

*Sauvagesia rhodoleuca* (Diels) M.C. E. Amara 2006 (Ochnaceae), also previously termed as *Sinia rhodoleuca* Diels, was the only representative of the genus *Sinia* endemic to China. But now it is incorporated into *Sauvagesia* based on the morphological characteristics of its flower and seed (Maria do Carmo 2006). *S. rhodoleuca* is a small shrub with natural distribution area limited to Guangdong and Guangxi, Southern China. Its rhizomes are very developed and can be used as medicine (Miao et al. 2020). However, due to deforestation, habitat destruction and the over-exploitation for medicinal purposes, the population size of *S. rhodoleuca* has decreased dramatically, causing it was listed as the state of ‘Vulnerable’ (UV) in The China Biodiversity Red List: Higher Plants (Wang and Xie 2004). Moreover, *S. rhodoleuca* has also been categorized as a Grade I National Key Protected Wild Pant (The State Forestry Bureau 1999). Given that genetic information is of great significant in species conservation, we here first assembled and characterized the complete chloroplast genome sequence of *S. rhodoleuca* using the Illumina paired-end sequencing data, which was registered into the NCBI with the BioProject codes PRJNA721244 and accession number MW772237.

Samples of *Sauvagesia rhodoleuca* was collected from Heishiding (Guangzhou, China, 23°27′7.20″ N, 111° 53′13.2″ E). Genomic DNA was extracted from the silica-dried leaves with modified CTAB method (Doyle and Doyle 1987) and was then sequenced on the BGISEQ-500 platform at Beijing Genomics Institute (Shenzhen, China). The specimen and DNA were deposited in the Herbarium of Wenzhou University (WZU) (contact Yonghua Zhang, zhangyhua@wzu.edu.cn) under the same voucher number (*Yonghua Zhang 20170514zyh02*). Finally, about 3.0 Gb clean data was assembled into complete chloroplast genome by using the Getorganelle v1.7.4 pipeline (Jin et al. 2020). Gene annotation was performed using the GeSeq (Tillich et al. 2017) and manually checked with the start/stop codons in Geneious v9.0.2 (http://www.geneious.com).

The chloroplast genome of *Sauvagesia rhodoleuca* was 157,300 bp in length and displayed a typical quadripartite structure of angiosperms, consisting of a large single-copy region (LSC, 86,021 bp), a pair of inverted repeat regions (IR, 26,571 bp) and a small single-copy region (SSC, 18,137 bp). The total GC content was 36.4%, whereas the GC contents in the LSC, SSC, and IR regions were 34.0%, 30.2%, and 42.5%, respectively. The chloroplast genome of *S. rhodoleuca* encoded 133 genes, of which 21 were duplicated in the IR regions. The remaining 112 unique genes included 80 protein-coding genes, 28 tRNA genes and 4 rRNA genes. Ten protein-coding genes and six tRNA genes contained one single intron, whereas two genes (*ycf3, clpP1*) had two introns.

To confirm the phylogenetic location of *Sauvagesia rhodoleuca*, we performed a phylogenetic analysis based on the maximum likelihood (ML) method implemented in RaxML (Nguyen et al. 2015). Because there is no published chloroplast genome in the family Ochnaceae and only one species *Medusagyne oppositifolia* has 44 complete chloroplast genes (Xi et al. 2012), we extracted the 44 genes from chloroplast genomes of other 16 species in the order Malpighiales. In addition, *Vitis vinifera* was used as outgroup. The sequences of the 44 genes from a total of 17 species were aligned by MAFFT version 7 software (Katoh and Standley 2013) and then used for the tree construction. Support for the ML tree was inferred by bootstrapping with 1000 replicates. Phylogenetic analysis results strongly supported that *S. rhodoleuca* was closely related to *M. oppositifolia* (Figure 1). This paper was the first to report the chloroplast genome of the family Ochnaceae and will provide useful genetic information for further study on genetic diversity and conservation of Ochnaceae species.

**Figure 1.**
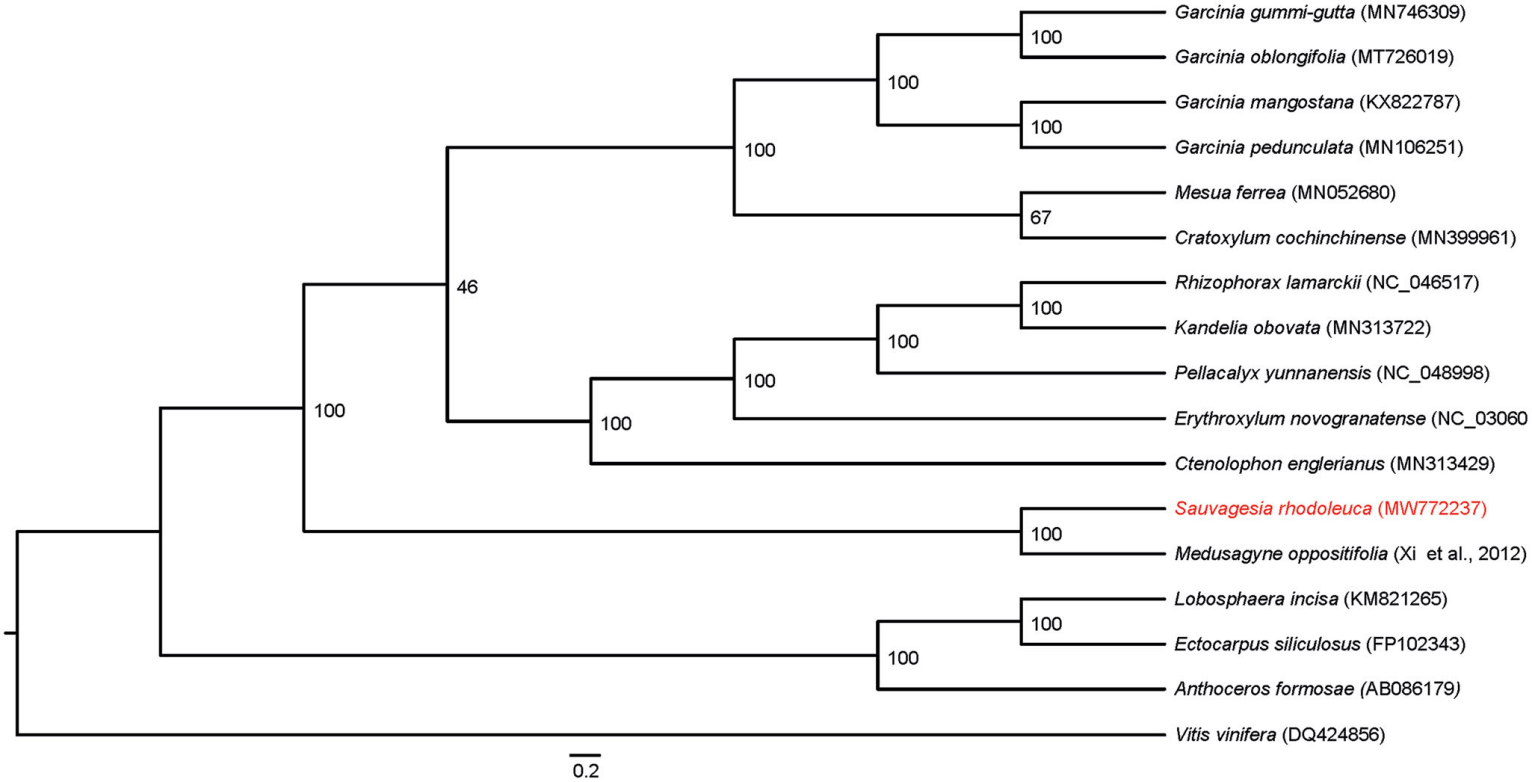
Phylogenetic tree reconstruction of 17 taxa of Malpighiales based on 44 chloroplast genes using ML method. Relative branch lengths are indicated. Numbers near the nodes represent ML bootstrap value.

## Data Availability

The genome sequence data that support the findings of this study are openly available in GenBank of NCBI at (https://www.ncbi.nlm.nih.gov/) under the accession no. MW772237. The associated BioProject, SRA, and Bio-Sample numbers are PRJNA721244, SRX10576401, and SAMN18712077, respectively.
